# Comparison of different surgical methods and strategies for inguinal lymph node dissection in patients with penile cancer

**DOI:** 10.1038/s41598-022-06494-z

**Published:** 2022-02-15

**Authors:** Yanxiang Shao, Xu Hu, Shangqing Ren, Duwu Liao, Zhen Yang, Yang Liu, Thongher Lia, Kan Wu, Sanchao Xiong, Weixiao Yang, Shuyang Feng, Yaohui Wang, Xiang Li

**Affiliations:** 1 Department of Urology, Institute of Urology, West China Hospital, Sichuan University, 37 GuoXueXiang, Chengdu, 610041 People’s Republic of China; 2 Robotic Minimally Invasive Surgery Center, Sichuan Academy of Medical Sciences and Sichuan Provincial People’s Hospital, Chengdu, People’s Republic of China; 3 Department of Urology, Nuclear Industry 416 Hospital, Chengdu, People’s Republic of China; 4 Department of Urology, Chengdu Second People’s Hospital, Chengdu, People’s Republic of China

**Keywords:** Cancer therapy, Urological cancer, Urology

## Abstract

To compare the clinical feasibility and oncological outcome of different surgical techniques for inguinal lymphadenectomy (ILND) in patients suffering from penile cancer. This study included data from 109 cN_0–2_ patients diagnosed with penile cancer who received ILND. 80 laparoscopic ILND were performed on 40 patients, while 138 open surgeries were performed on 69 patients. Perioperative complications and prognosis were compared between different surgical techniques. Compared with the open surgery group, the laparoscopy group had a shorter hospital stay (8.88 ± 7.86 days vs. 13.94 ± 10.09 days, *P* = 0.004), and a lower wound healing delay rate (8.75% vs. 22.46%, *P* = 0.017), but also had longer drainage time (10.91 ± 9.66 vs. 8.70 ± 4.62, *P* = 0.002). There were no significant differences in terms of other intraoperative parameters, complications, and survival between open and laparoscopic group. Compared with saphenous vein ligated subgroup, preserved subgroup showed no significant reducing of complication rate. There was no significant difference among complication between different open surgery subgroup. Immediate ILND showed no prognostic advantage over delayed ILND regardless of clinical lymph node status. Compared with open surgery, the minimally invasive ILND technique has similar oncological efficiency and a lower complication rate. Saphenous vein preservation has limited value in reducing complications. Delayed lymphadenectomy might be a more reasonable option for ILND.

## Introduction

As a rare genitourinary tumor, penile cancer has an overall incidence of < 1 in 100,000 males in American and European countries^1,2^. However, this number is increasing in many developing countries, including those in Africa, South America, and South East Asia; collectively, the incidences of penile cancer in these regions account for 1–2% of all malignant tumors in men^3^. The inguinal lymph nodes (ILNs) are the most common metastatic site for penile cancer, a condition that is always associated with a poor prognosis^4,5^. Inguinal lymph node dissection (ILND) is the standard treatment protocol for penile cancer in patients with high-risk disease, palpable and enlarged ILNs, or pathologically confirmed ILN metastasis^6^.

Although ILND can help tumor grading and reduce the risk of mortality, this technique is also associated with a high incidence of complications, with an incidence as high as 70%^7,8^. The most common complications are wound-related problems (infection, delayed healing, and skin necrosis) and lymph-related problems (lymphocele, lymphatic fistula, and lower limb edema). Many techniques have been developed to reduce such complications including modifications in the field of dissection and incision methods, preservation of the great saphenous vein (GSV), endoscopic surgery, and delayed ILND^9–11^. However, the standard surgical protocol remains controversial^3^. The aim of our study was therefore to retrospectively acquire data from patients with penile cancer undergoing ILND at the West China Hospital, and to evaluate different surgical techniques and strategies with special reference to complications and prognosis.

## Materials and methods

### Research population

This study included patients who were diagnosed with penile squamous cell carcinoma (PSCC) and treated in the Department of Urology at West China Hospital, Sichuan University, between August 2008 and September 2020. Regardless of partial or radical penectomy received, ILND was recommended to those patients who had been diagnosed as pT1G2 or higher stages, and those with palpable inguinal lymph node, with pros and cons of immediate or delay ILND be fully informed. Decision about the choose of open or minimally invasive surgery was made by patients themselves after fully informed pros and cons of these two different operative methods. For delayed ILND patients, they were follow-up by clinical examination in every 3–6 months, and when enlargement of inguinal lymph node was found, ILND was recommended to patients again. We excluded patients with a fixed inguinal nodal mass, or pelvic metastasis, that were identified prior to ILND (cN3); those with an Eastern Cooperative Oncology Group score exceeding 1; and those who were not willing to provide clinical information for our research. All patients provided signed consent and were fully informed before their clinical data were collected. We finally included 109 patients with PSCC treated with ILND. The whole retrospective research process was approved before research conduct, and completed under the supervision and guidance of West China Hospital of Sichuan University Biomedical Research Ethics Committee.

### Surgical procedures

#### Open surgery (O-ILND)

**Figure 1:**
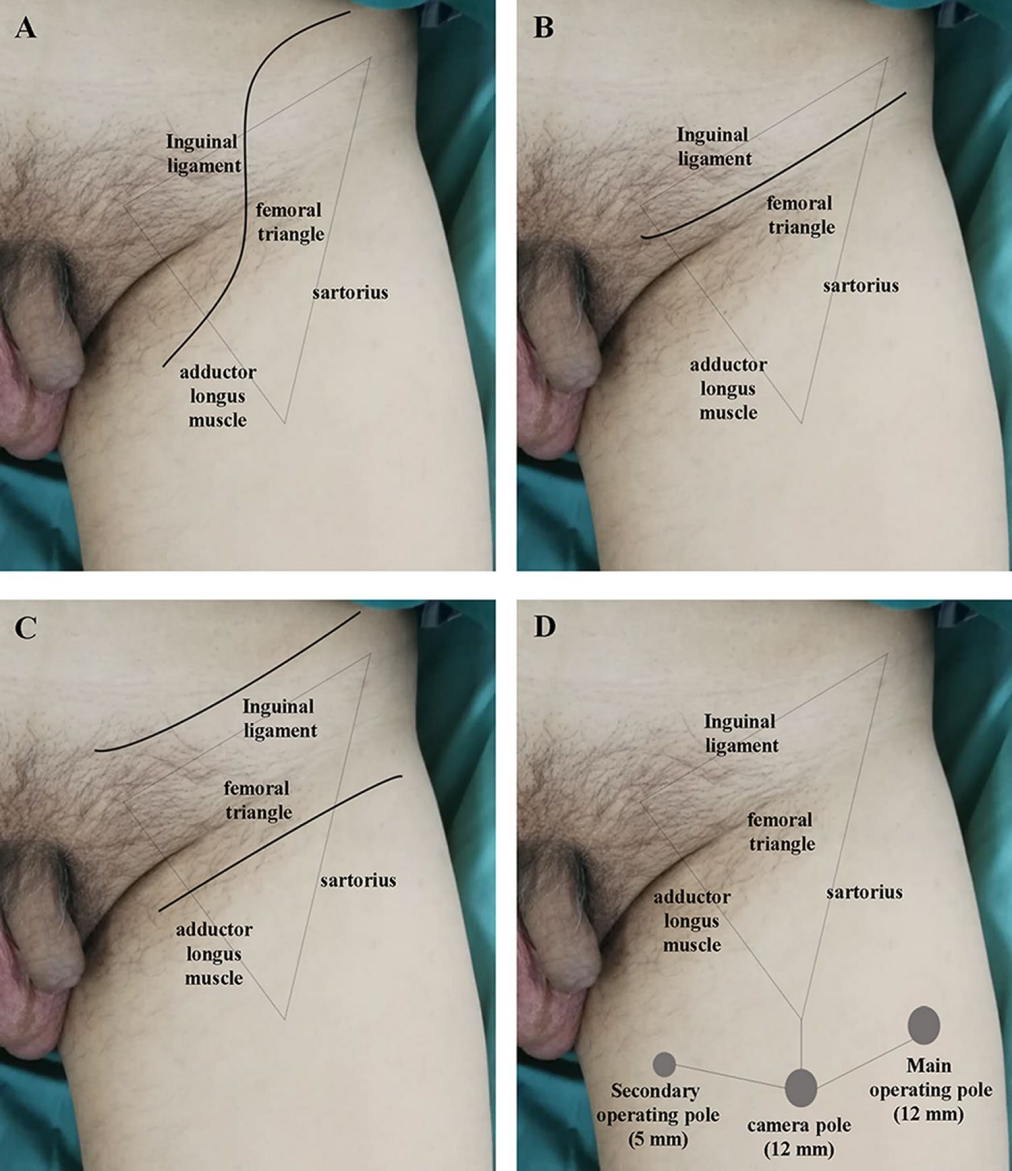
Incisions for different surgical techniques (left side view). (**A**) S-shaped incision; (**B**) Single oblique incision; (**C**) skin bridge incision (Fraley incision); (**D**) Trocar placement of laparoscopic inguinal lymphadenectomy.

Bilateral O-ILND was performed with the patient in a supine position with their legs placed in the frog-leg position. Three skin incision methods were used: an s-shaped incision, a single oblique incision, and a skin bridge incision (the Fraley incision)^12^ (Fig. 1A,B,C). The s-shaped incision was commenced in the lateral margin, approximately 3 cm above the anterior superior iliac spine, and were terminate in the medial margin approximately 2 cm below the external ring. The single oblique incision was commenced approximately 2 cm superior to the inguinal arch and was extended for 12–18 cm. The skin bridge incision was performed in accordance with the method described previously by Fraley^12^: two incisions were created 15 cm parallel to the inguinal ligament; these incisions were created 4 cm above and 2 cm below the inguinal ligament. Dissection boundaries were defined in accordance with the modified procedure reported by Catalona et al.^9^. In brief, the adduction muscle was defined as the medial margin while the femoral artery was defined as the lateral margin. The spermatic cord was used as the upper boundary while the oval fossa was used as the lower boundary. Adipose and lymphatic tissue was freed in one piece from the top down and from the periphery to the center. The GSV was preserved if skeletonized feasibly and no enlarged lymph node remained, with complete ligation of its branches. The sheath of the femoral artery and vein were then incised below the femoral triangle in order to remove the femoral lymph nodes. If potential metastatic femoral lymph nodes were observed, then pelvic lymph node dissection (PLND) was performed. This involved removal of the pelvic fascia, the vagina vasorum of the common and external iliac vessels, the peripheral lymph nodes, and the adipose tissue below the aortic bifurcation. Finally, a subcutaneous drainage-tube, connected to a negative pressure suction bottle, was placed in the dissected area and the incision was sutured in a layer-by-layer fashion.

#### Laparoscopic surgery (L-ILND)

For bilateral L-ILND, we used the same operative position and dissection boundaries as for open surgery. First, we made a 1.5 cm incision approximately 2 cm below the apex of the femoral triangle to create a camera port. Using a finger to probe through the incision, we created a blunt separation above the Scarpa fascia; this separation was extended upwards and laterally as far as possible. Two working ports (12 mm, right hand port; 5 mm, left hand port) were then placed approximately 6 cm medial and lateral to the camera port; this was carried out under finger perception. The triangle formed by these three pointed towards the target dissection area (Fig. 1D). Next, a 12 mm trocar was placed in the camera port. After pneumoperitoneum space established, a harmonic scalpel was used to dissect the superficial lymph node and ligated the branch of the saphenous vein. If preservation of the GSV was anatomically difficult, ligated using an absorbable ligating clip. Then, we opened the femoral canal sheath and removed the deep lymph nodes. In cases requiring PLND, we collected the PLNs transabdominally, with camera port just below the naval and two working ports at both side of the mid of the line between naval and the anterior superior iliac spine. After removing excised tissue, negative pressure drainage-tubes in the surgical area were placed through the lateral working port. Finally, we closed all incisions.

#### Postoperative treatment

Most patients were confined to their beds for 3–4 days after surgery. During these times, we encouraged patients to move their lower limbs and to wear stretch socks. Patients were also recommended to resume their diet after 6 to 8 h after surgery. A sandbag weighing 1.5 kg was placed above the wound in order to prevent subcutaneous lymphatic effusion, and a drainage-tube was used to maintain negative pressure in the inguinal region. Usually, these tubes were removed if the drainage volume was less than 50 ml over a 48-h period. When patients recovered with good diet and activity, with no serious complications observed, they would be transferred to community for further hospitalization and rehabilitation. Patients were followed-up by clinical examination in one month after surgery, then every 3 months in the first year and 6 months thereafter. Ultrasonography of the groin was performed every 6 months for the first 2 years after surgery.

#### Data collection

We collected a range of clinical and pathological data, including age, smoking history, data arising from the physical-examinational data prior to penile surgery, penile cancer T stage and nuclear grade, the time interval between penile surgery and ILND, physical-examinational data prior to ILND, ILND methods, length of ILND procedure, operative blood loss, drainage-tube removal time, length of postoperative hospital stay (from the date of operation to discharge from hospital or community rehabilitation center), complications, lymph node harvest, and pathological N stage (pN). T stage, N stage, and nuclear grading, were adjusted according to the 2016 Union for International Cancer Control (UICC) TNM classification for penile cancer^13^. Wound healing delay was defined as a healing period > 14 days, and delay of drainage-tube removing was defined as longer than 10 days. Clavien–Dindo Complication Grading (I-IV) was used to judge postoperative complications.

#### Statistical methods

One-way analysis of variance (ANOVA) and the chi-squared test were used to compare data between different groups. Fisher’s exact tests, along with Kruskal–Wallis and Wilcoxon tests, were used to analyze categorical and continuous variants. A time interval between penile surgery and ILND that was less than 1 month was defined as an ‘immediate’ operation, while longer time intervals were defined as a ‘delayed’ operation. The start for survival research was defined as the timepoint at which ILND was performed, while the endpoint was the timepoint local progression and distant metastasis were detected, or death occurred. Progression-free survival (PFS) and overall survival (OS) were estimated using the Kaplan–Meier method. Univariate Cox regression analyses were performed to determine the clinicopathological parameters associated with the survival of PSCC patients. Statistical analyses were performed using IBM SPSS Statistical software version 25 (Chicago, USA), and *P* < 0.05 denoted statistical significance.

### Declaration of ethics approval and consent to participates

The authors are accountable for all aspects of the work in ensuring that questions related to the accuracy or integrity of any part of the work are appropriately investigated and resolved. This retrospective study followed the guideline of the Declaration of Helsinki. Patients and their authorized family members had been fully informed before follow-up work was performed, with informed consent signed. The whole process was completed under the supervision and guidance of West China Hospital of Sichuan University Biomedical Research Ethics Committee.

### Consent for publication

Consent for publication was obtained from all participants.

## Results

Clinicopathological data derived from 109 cases of patients with PSCC and treated by ILND are shown in Table 1. Mean age (± standard deviation) was 50.75 ± 12.52 years, and the median follow-up time was 43.04 months (interquartile range, IQR: 14.75–86.84 months). Overall, 80 and 138 separate L-ILND and O-ILND procedures were performed on these patients (40 and 69 patients underwent L-ILND and O-ILND, respectively). None of the patients were diagnosed with pathological lymph node stage 3 (pN3). There were no significant differences between the two groups of patients with regards to the baseline data (Table 1).

**Table 1:** Clinicopathological data relating to 109 patients with penile cancer.

Variants	L-ILND	O-ILND	*P* value	Total
**Age**			0.801	
< 60 years-of-age	31	52		83
≥ 60 years-of-age	9	17		26
**Smoking**			0.363	
No	21	30		51
Yes	19	39		58
**Grade**			0.572	
Well differentiated	13	18		31
Moderately differentiated	19	30		59
Poorly Differentiated/undifferentiated	8	11		19
**T stage**			0.173	
Ta/Tis/T1	16	16		32
T2	11	26		37
T3	13	27		40
T4	0	0		0
**cN**			0.370	
cN0	7	20		27
cN1	8	14		22
cN2	25	35		60
**ILND time**			0.496	
Immediate surgery	6	16		26
Delayed surgery	21	62		83
**pN**			0.131	
pN0	26	32		58
pN1	7	14		21
pN2	7	23		30
**Survival**			0.579	
Alive	33	52		85
Dead	7	17		24

*L-ILND* laparoscopic inguinal lymph node dissection, *O-ILND* open inguinal lymph node dissection.

*P* values refer to differences in the indicated parameters when compared between the O-ILND and L-ILND groups.

Perioperative data are shown in Table 2. For the comparison between laparoscopy and open surgery, intraoperative blood loss was statistically lower in L-ILND group, but with no clinical significance (23.76 ± 13.02 ml vs. 39.67 ± 22.79 ml, *P* = 0.001). Longer drainage time were shown in laparoscopic group (10.91 ± 9.66 days vs. 8.70 ± 4.62 days, *P* = 0.002). Hospital stay was significantly longer in duration for patients undergoing open surgery (8.88 ± 7.86 days vs. 13.94 ± 10.09 days, *P* = 0.004). For comparison of GSV preserved and ligated group, we see that GSV preserving might prolong operation time, but might help to remove drainage-tube earlier, although these differences were not significant (*P* = 0.943 and 0.873 respectively). In terms of lymph nodes harvest, although the mean number of open surgery group was slightly higher than endoscopic group, the difference was not significant (*P* = 0.110). No significant difference among lymph nodes harvest was also demonstrated between GSV ligated and preserved group (*P* = 0.369).

**Table 2:** Perioperative details for ILND treated patients.

Variants	L-ILND	O-ILND	*P* value	GSV ligated	GSV preserved	*P* value	Total
Operation time (min)	60.44 ± 22.75	64.78 ± 29.47	0.102	61.61 ± 27.37	66.26 ± 26.86	0.943	63.19 ± 27.22
Intraoperative blood loss (ml)	23.76 ± 13.02	39.67 ± 22.79	0.001	33.48 ± 23.65	34.51 ± 15.40	0.071	33.83 ± 21.18
Drainage time (days)	10.91 ± 9.66	8.70 ± 4.62	0.002	9.87 ± 7.33	8.80 ± 6.20	0.873	9.51 ± 6.97
**LN harvest (number of removed lymph nodes)**							
ILN	6.70 ± 3.55	7.39 ± 3.91	0.125	7.05 ± 3.68	7.30 ± 4.01	0.284	7.13 ± 3.79
PLN	3.17 ± 2.41	3.06 ± 2.33	0.906	2.88 ± 2.20	3.53 ± 2.59	0.358	3.08 ± 2.33
Total	7.28 ± 4.06	8.73 ± 4.50	0.110	8.17 ± 4.19	8.24 ± 4.79	0.369	8.20 ± 4.39
Hospital stay (days)	8.88 ± 7.86	13.94 ± 10.09	0.004	–	–	–	11.89 ± 9.58

*L-ILND* laparoscopic inguinal lymph node dissection, *O-ILND* open inguinal lymph node dissection, *GSV* great saphenous vein, *PLND* pelvic lymph node dissection. Operative time, blood loss, drainage time, GSV preservation, PLND performed, and the number of LNs harvested were analyzed for different surgical types (80 L-ILND cases and 138 O-ILND cases, respectively). Postoperative hospital stay was analyzed for patients treated by different techniques (40 cases of L-ILND and 69 cases of O-ILND, respectively).

Complications were also analyzed (Table 3). In comparison of L-ILND and O-ILND, results indicated that the incidence of delayed wound-healing was significantly higher for open surgery (22.46% vs. 8.75%, *P* = 0.017). There were no significant differences in the rates of wound infection, dehiscence, or skin flap necrosis, when compared between open and laparoscopic groups (*P* > 0.05). Similarly, there was no significant difference in terms of lymph-related complications (drainage-tube removing delay, lymphocele, and lower limb edema). Only one grade III complication occurred in a patient treated by L-ILND (skin necrosis during the first thirty days after surgery). There was no significant difference between the two surgical types with regards to grade I or II complications. Besides, for GSV preserved versus ligated groups, we see the retention of the GSV didn’t significantly reduce the complication rate (*P* > 0.05).

**Table 3:** Complications reported in the minimally invasive and open groups of patients.

Object	Complications (number)	L-ILND	O-ILND	*P* value	GSV ligated	GSV preserved	*P* value
		n = 80	n = 138		n = 144	n = 74	
Total 218 surgeries	Wound infection	2	9	0.324	7	4	1.000
	Wound healing delay	7	31	0.017	28	10	0.274
	Wound dehiscence	0	2	0.787	2	0	0.196
	Skin flap necrosis	1	3	1.000	3	1	1.000
	Drainage-tube removing delay	22	30	0.336	33	19	0.651
	Lymphocele	11	18	0.882	18	11	0.626
	Lower limb edema	17	32	0.741	28	21	0.135
Total 109 patients		n = 40	n = 69		NA	NA	NA
	Scrotal edema	2	5	0.541	NA	NA	NA
	Urine infection	3	5	1.000	NA	NA	NA
Clavien grade	No complication	19	25	0.367	NA	NA	NA
	Grade I	16	30	0.921	NA	NA	NA
	Grade II	4	14	0.260	NA	NA	NA
	Grade III	1	0	–	NA	NA	NA
	Grade IV	0	0	–	NA	NA	NA

*L-ILND* laparoscopic inguinal lymph node dissection, *O-ILND* open inguinal lymph node dissection, *n* number.

Subgroup analysis of endoscopic and open groups were shown in Table 4. In L-ILND group, GSV preservation was performed in 40% of surgeries. However, this technique not only lead no fewer complication, but had a higher probability of delay drainage-tube removing and lower limb edema (*P* = 0.042 and 0.005 respectively). In O-ILND group, 88, 48 and 2 surgeries were performed as skin bridge, single oblique and S-shaped separately; 30.43% of them had GSV preserved. In this subgroup, either retaining the GSV or using a certain open procedure didn’t significantly reduce the complication rate (*P* > 0.05).

**Table 4:** Subgroup analysis Complications reported in the patients receiving GSV preservation and GSV ligation.

Complications (number)	L-ILND			O-ILND			O-ILND			*P* value
Subgroups	GSV ligated	GSV preserved	*P* value	GSV ligated	GSV preserved	*P* value	S-shaped	Single oblique	Skin bridge	
	n = 48	n = 32		n = 96	n = 42		n = 2	n = 48	n = 88	
Wound infection	1	1	1.000	6	3	1.000	0	4	5	0.755
Wound healing delay	5	2	0.696	23	8	0.659	0	9	22	0.713
Wound dehiscence	0	0	NA	2	0	1.000	0	0	2	0.553
Skin flap necrosis	0	1	0.400	3	0	0.553	0	1	2	1.000
Drainage-tube removing delay	9	13	0.042	24	6	0.185	0	11	19	1.000
Lymphocele	5	6	0.333	13	5	1.000	1	3	14	0.080
Lower limb edema	5	12	0.005	23	9	0.829	2	10	20	0.082

*GSV* great saphenous vein, *n* number, *L-ILND* laparoscopic inguinal lymph node dissection.

**Figure 2:**
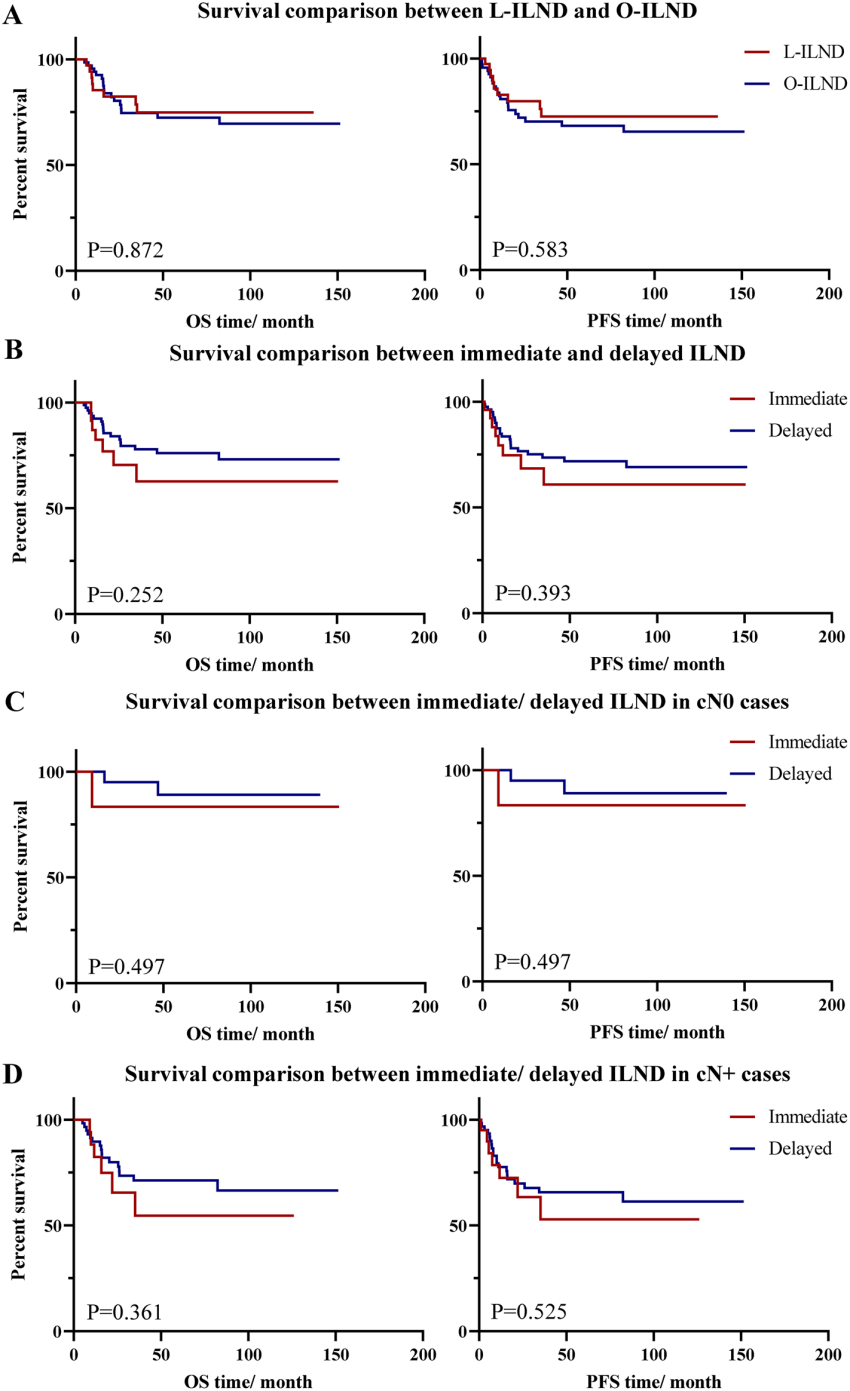
Survival comparison of different surgical subgroups. (**A**) Survival comparison between L-ILND and O-ILND; (**B**) Survival comparison between immediate and delayed ILND; (**C**) Survival comparison between immediate/ delayed ILND in cN0 cases; (**D**) Survival comparison between immediate/ delayed ILND in cN + cases. L-ILND: laparoscopic inguinal lymph node dissection; O-ILND: open inguinal lymph node dissection; cN0: clinical N stage 0; cN + : clinical N stage positive.

Prognosis of patients after ILND were analyzed. During follow-up, 24 patients dead and all caused by PSCC. Survivorship analysis showed that the 2- year and 5-year survival rates for PFS, OS were 74.8% and 69.7%; 81.2% and 73.3%. 26 patients underwent immediate ILND and other 83 patients choose delayed operations (cN + patient number were 18 and 61 separately). Kaplan–Meier analysis and Log-rank test demonstrated that no significant difference in OS and PFS between endoscopic and open ILND groups, or immediate and delayed ILND groups (Fig. 2A,B). Even in subgroup analysis of cN0 or cN + , there is no significant difference of prognosis between immediate and delayed ILND (Fig. 2C,D).

## Discussion

When treating PSCC, there are two central questions that surgeons must consider: whether and how to perform ILND. On the one hand, ILND can enable the oncological status to be staged and allow removal of the potential metastatic site; on the other hand, side effects appear to be inevitable for ILND. Consequently, surgeons always seek to balance both of these aspects. In this study, we investigated and compared the efficacy of optimizing surgical incision, preserving the GSV and the application of endoscopy using long-term follow-up data and outcome from different surgical timings. We hoped that this analysis might answer the two central questions described above.

There is still debate with regards to the choice of incision. A range of incision methods have been proposed, including the single oblique incision, the arc-sharped incision, and the s-sharped incision. The skin bridge technique was first reported by Fraley in 1972, and involves two incisions that are made parallel to the upper and lower region of the groin; this technique provides better exposure of the dissection area^12^. According to a recent study involving 75 patients, the skin bridge technique led to successful treatment in 68% of cases without complications^14^. Of all the open ILND methods available, the skin bridge technique was the mostly commonly used in our series of patients; however, this technique did not appear to be associated with any specific advantage. In our experience, complications are inevitable in some patients owing to the poor blood supply to the skin in the inguinal region. The skin bridge covers the femoral vessels, thus preventing these fragile structures from being exposed to the air in the event of complications such as skin necrosis, wound infection, and dehiscence. For patients with poor blood supply below the flap, sartorius transposition may be a way to reduce local wound complications^15^.

In the conventional ILND technique, the GSV is normally ligated; however, this practice blocks lymphovascular circulation in the lower limbs and may lead to a range of skin-related problems, including lower limb lymphedema^16^. GSV preservation was first reported by Catalona et al. to minimize complications^9^. A recent meta-analysis of vulval malignancy has shown that this technique could reduce the rate of lymphedema, wound necrosis, and acute cellulitis in cases experiencing open ILND^17^. In our present study, the advantages associated with GSV preservation remain controversial. In our experience, GSV preserved operation always need larger extent of dissection and more use of energy instrument to ensure complete removal of lymph nodes during the skeletal process of vein. Whether this reduced the benefits of preservation of GSV still needs further research to determine. Large scale prospective study will be helpful to reflect the benefit of GSV preservation for the ILND of penile cancer.

Previous research claimed that L-ILND should reduce perioperative morbidity^18–20^. A recent meta-analysis, involving 10 studies and 307 patients, compared open and endoscopic ILND cases and found that the minimally invasive group had reduced levels of intraoperative blood loss, shorter hospital stays, shorter drainage times, and reduced rates of wound- and lymphatic-related complications; however, the number of harvested lymph nodes was slightly lower than for the open surgery group^21^. Our current results are partially consistent with these previous results. As for the LNs harvest, it is possible for urologist to dissect less lymph nodes than open surgery in the early stage of laparoscopic surgery. With the growth curve into the plateau, the LNs harvest should go equal to that for open surgery, since there is no difference for the scope of dissection regardless of minimally or open procedures. At the same time, we believe that even in the era of minimally surgery, surgeons must gain expertise in all of these techniques and develop an individualized treatment plan for their patients, because open surgery is still irreplaceable in some cases (for example, due to skin damage, extreme obesity, or emaciation).

The number of dissected lymph nodes is an important index that can be used to evaluate the oncological efficacy of various surgical methods^22–24^. Kumar et al. reported that videoendoscopic ILND was able to harvest significantly more ILNs than open surgery (9.36 vs. 7.11; *P* = 0.013)^19^. A meta-analysis by Hu et al. further demonstrated that significantly larger number of ILNs were found in the open group than the endoscopic group (162 patients in total; *P* = 0.030)^21^. In our present research, the mean number of LNs harvested was higher in the group of patients receiving open surgery, although the difference was not statistically significant. Further studies are now needed to investigate oncological outcomes in greater detail.

There is still no specific conclusion with regards to the relative survival rate of patients following open and endoscopic surgery. Although Shi et al. and Wang et al. both reported higher recurrence rates for patients treated with L-ILND (14.29% vs. 8.33% and 11.11% vs. 6.25% for open versus endoscopic groups)^18,25^, this could not reflect prognostic differences due to the limited sample sizes and follow-up length. According to early-stage records of open surgery, the 5-year survival rate after ILND ranged from 29 to 86%^26,27^. In an O-ILND series of 75 patients reported in 2018, 73.3% of patients survived and remained disease-free, while 12% died^14^. However, survival analysis could not be performed because of the limited follow-up period (median length: 17.64 months). In our study, which featured long-term follow-up, we found no significant difference between the open and L-ILND subgroups with respect to survival. In fact, we considered that minimally invasive surgery, as a replication of open surgery (but using endoscopy), should theoretically have a similar survival benefit to the open technique as long as a sufficient number of LNs can be removed from the target area.

ILND can enable the staging of oncological status and the removal of potential metastatic sites. Even so, unnecessary ILND should undoubtedly be reduced since inevitable complications. Surgeons cannot decide on ILND by clinical N stage alone, since analysis revealed a false positive rate that ranged from 8 to 65% and a false negative rate that ranged from 2 to 100%^28^. Our current work showed that in either for cN0 or cN + cases, immediate ILND did not provided a survival advantage. It’s more reasonable to choose active surveillance and delayed ILND after penile surgery since unnecessary ILND-related complications can be avoided.

Although the limitations associated with retrospective studies are unavoidable and many issues have yet to be clarified, the limited body of literature that is currently available in the era of minimally invasive surgery is becoming increasingly important, particularly as the number of cases involving penile cancer is increasing globally. To our knowledge, our research study is the largest comparative study in recent years to focus on different surgical methods and techniques over a long-term period and with a complete set of follow-up data. Future research should aim to increase case numbers and include long-term follow-up data with particular emphasis on oncological outcome.

## Conclusion

L-ILND is associated with less hospital stay and a lower rate of wound-healing delay when compared with O-ILND. There were no significant differences between these two groups with regards to the number of LNs harvested or the total rate of complications. Preservation of the GSV might not help to reduce complication rate no matter in L-ILND or O-ILND. Compared with delayed ILND, immediate ILND didn’t show any prognostic advantage.

## Supplementary Information

Supplementary Information 1.

Supplementary Information 2.
