# Supplementary material for: Comparison of different surgical methods and strategies for inguinal lymph node dissection in patients with penile cancer
Source: Sci Rep. 2022 Feb 15;12:2560. doi: 10.1038/s41598-022-06494-z (PMC8847572; doi:10.1038/s41598-022-06494-z)
Supplement: Supplementary file 2 — Supplementary Information 2. [file 41598_2022_6494_MOESM2_ESM.docx]

**Original articles**

**Comparison of different surgical methods and strategies for inguinal lymph node dissection in patients with penile cancer**

Yanxiang Shao, MD^1^*, Xu Hu, MD^1^*, Shangqing Ren, MD^1,2^*, Duwu Liao, MD^1,3^, Zhen Yang, MD^1,4^, Yang Liu, MD^1^, Thongher Lia, MD^1^, Kan Wu, MD^1^, Sanchao Xiong, MD^1^, Weixiao Yang, MD^1^, Shuyang Feng, MD^1^, Yaohui Wang, MD^1^, Xiang Li, MD, PhD^1^

^1^Department of Urology, Institute of Urology, West China Hospital, Sichuan University, Chengdu P.R. China.

^2^Robotic Minimally Invasive Surgery Center, Sichuan Academy of Medical Sciences & Sichuan Provincial People's Hospital, Chengdu P.R. China.

^3^Department of Urology, Nuclear Industry 416 Hospital, Chengdu P.R. China.

^4^Department of Urology, Chengdu Second People’s Hospital, Chengdu P.R. China.

*These authors contributed equally to this work.

**ORIGINAL DATASET LEGENDS**

This original dataset included all of the data used for current study. ILN: Inguinal lymph node; cN: clinical N stage; GSV: great saphenous vein; LN: lymph node; PLN: pelvic lymph node; EBL: estimated blood loss.
